# Get Moving! Increases in Physical Activity Are Associated With Increasing Functional Connectivity Trajectories in Typically Aging Adults

**DOI:** 10.3389/fnagi.2020.00104

**Published:** 2020-04-28

**Authors:** Karen A. Dorsman, Sophia Weiner-Light, Adam M. Staffaroni, Jesse A. Brown, Amy Wolf, Yann Cobigo, Samantha Walters, Joel H. Kramer, Kaitlin B. Casaletto

**Affiliations:** ^1^Department of Psychiatry, Division of Psychology, University of Texas Southwestern Medical Center, Dallas, TX, United States; ^2^Weill Institute for Neurosciences, Memory and Aging Center, University of California, San Francisco, San Francisco, CA, United States; ^3^Global Brain Health Institute, University of California, San Francisco, San Francisco, CA, United States

**Keywords:** brain health, physical activity, neuroplasticity, neuroimaging, functional MRI, functional connectivity

## Abstract

**Background**: Physical activity closely relates to cognition and brain structure as we age. However, the neural mechanisms underlying this relationship in humans remain less clear. Functional connectivity (FC), measured by task-free functional MRI (tf-fMRI) is a dynamic marker of network activity and may be a sensitive indicator of the brain’s response to exercise over time. We aimed to test the longitudinal relationship between physical activity and FC trajectories in functionally normal older adults.

**Methods**: Two hundred and twelve functionally normal, longitudinally-followed older adults completed the Physical Activity Scale for the Elderly (PASE) and tf-fMRI scans at each visit [mean = 1.5 visits (range:1–3)]. We studied FC of the default mode network (DMN), frontal-parietal (FP), subcortical networks (SubCort), and frontal-subcortical inter-network connectivity (FS), given that previous studies implicate these regions in age-related changes. Linear mixed-effects models examined the relationship between within-person changes in PASE and FC (in SD units), covarying for age, sex, education and systemic cardiovascular risk factors (heart rate, BMI and systolic blood pressure). We additionally examined models covarying for DTI fractional anisotropy (FA) and mean diffusivity (MD) of tracts underlying networks of interest, as a marker of cerebrovascular disease. Furthermore, we examined the longitudinal relationship between PASE and neuropsychological trajectories.

**Results**: In our first model, within-subject increases in physical activity tracked with increasing SubCort (*β* = 0.33, *p* = 0.007) and FS inter-network (*β* = 0.27, *p* = 0.03) synchrony, while between-subject parameters did not reach significance (*β* = −0.042 to −0.07, *p*s > 0.37). No significant longitudinal associations were observed between PASE and DMN (*β* = −0.02 *p* = 0.89) or FP networks (*β* = 0.15, *p* = 0.23). Adjusting for markers of cerebrovascular health (FA/MD) did not change estimated effects (SubCort: *β* = 0.31, *p* = 0.01, FS inter-network: *β* = 0.28, *p* = 0.03). Associations between changes in physical activity and neuropsychological trajectories were small (*β* = −0.14 to 0.002) and did not reach statistical significance (*p*-values >0.42).

**Conclusions**: Our findings suggest that changes in exercise over time are specifically associated with frontal-subcortical processes in older adults. This relationship appears to be independent of cardio- or cerebrovascular disease, possibly driven by a more direct neural response to exercise.

## Introduction

Cognitive decline is a public health issue of global proportions, as rates of dementia are rapidly increasing around the world. Currently, about 50 million people are living with dementia, and this number is projected to increase threefold in the next 30 years (Adams et al., 2005; Patterson, 2018). The burden of cognitive decline is pervasive, affecting individuals, families, communities, and society at large. As such, it is increasingly important to identify protective factors of cognitive aging decline, so that empirically-based primary and secondary prevention strategies can be developed. Physical activity has been identified as one of the most potent modifiable lifestyle factors to be associated with brain health in aging. While the evidence is mixed, with some studies suggesting that exercise may not benefit cognitive outcomes (van Uffelen et al., 2008; Young et al., 2015; Zuniga et al., 2016; Lamb et al., 2018), meta-analytic reviews and several large-scale epidemiological works have demonstrated that engaging in regular physical activity is consistently linked to a reduced risk of cognitive impairment and dementia (Laurin et al., 2001; Young et al., 2015; Northey et al., 2018). For instance, individuals who engage in higher levels of physical activity are estimated to have an approximately 25% lower risk of developing dementia (Hamer and Chida, 2009), and greater physical fitness in midlife is associated with delayed onset of dementia by up to 9.5 years (Hörder et al., 2018). Furthermore, randomized controlled studies make a case for a causal relationship between physical activity and cognition (Erickson et al., 2011), encouraging the implementation of exercise-related intervention strategies across the life span. This is especially appealing because there are countless ways to move, and being active does not necessarily require special equipment, social support, or financial assets. Thus, no matter the socioeconomic status of a community or an individual, an exercise intervention may be attainable. As evidence shows that the rising rates of dementia are most prevalent in low- and middle-income countries (Patterson, 2018), the possibility of developing low-cost prevention strategies is of particular interest.

Although various studies have identified physical activity as a possible primary preventive protective factor for brain health (Yaffe et al., 2001; Blondell et al., 2014; Daskalopoulou et al., 2017), the mechanisms by which physical activity affect cognitive function are not fully understood. Until recently, it was thought that physical activity was beneficial to brain health by means of reducing the impact of known risk factors, such as cardiovascular and cerebrovascular disease, stroke or diabetes. However, there is a growing body of literature from human and animal studies that indicates that the benefits may be more direct, involving the promotion of synaptogenesis, neuroplasticity, and growth and survival of neurons, as well as the reduction of inflammation and stress (Macpherson et al., 2017; Mattson and Arumugam, 2018).

The field of cognitive aging is constantly seeking more reliable biomarkers that accurately reflect the brain’s functioning and can help us better understand the mediating pathways of protective and/or risk factors that impact brain structure and functioning. Task-free Functional Magnetic Resonance Imaging (tf-fMRI), also referred to as “resting-state fMRI,” is a widely-used tool to explore the integrity and function of large-scale brain networks. Functional connectivity (FC) is one factor that has been reported to be affected by the aging process. It is thought to reflect typical cognitive changes in aging (Onoda et al., 2012; Geerligs et al., 2015), including associations with episodic memory, processing speed and working memory (Andrews-Hanna et al., 2007; Siman-Tov et al., 2017; Staffaroni et al., 2018). Previous literature has documented disruptions in major large-scale networks during aging in the absence of disease; however, these findings have focused mostly on the default mode network (DMN) and its connections to other regions.

Previous studies on the effects of physical activity on the brain have typically focused on structural changes, and most are based on short-term interventional studies (Stillman et al., 2019). More recently, some interventional, tf-fMRI-based studies have shed light on how connectivity is affected by physical activity. For example, in a 12-week physical activity intervention, the relationship between the primary motor regions and the DMN were found to be associated with better motor performance (McGregor et al., 2018), and after 6 months of moderate- or high-intensity aerobic exercise, older adults with amnestic mild cognitive impairment (MCI) showed increased FC in the prefrontal cortex (Hugenschmidt et al., 2017). However, there is currently not sufficient longitudinal data to allow us to understand physical activity’s long-term effects on FC.

In the present study, we examined the longitudinal relationship between FC and self-reported changes in physical activity in community-dwelling older adults. Given that the DMN, the frontal-parietal network (FPN, also known as the central executive network), and the subcortical network (SN) are widely-examined networks that are associated with abilities such as introspection, executive function, and motor function, respectively, we focused our preliminary investigations on connectivity within these three networks. We hypothesized that an increase in exercise would relate to increased synchrony within these networks. By providing longitudinal data on the relationship between physical activity and the brain’s FC, we hope to inform how this widely-accessible modifiable lifestyle factor may impact brain health, and how FC could be used to track those changes.

## Materials and Methods

### Participants

In this study, we included 212 older adults who were longitudinally-followed in the Hillblom Healthy Aging Study at the UCSF Memory and Aging Center (see Table 1). The Hillblom Aging Network cohort consists of neurologically- and functionally-intact, community-dwelling older adults. The UCSF Committee on Human Research approved the study protocol and, per their guidelines, all subjects provided written, informed consent. During the initial screening visit, participants underwent a comprehensive neurological and neuropsychological clinical examination. An interdisciplinary team reviewed each case to confirm clinically-normative status. Subjects were assigned a clinical dementia rating (CDR) based on a semi-structured interview with a collateral source. The CDR is a global clinical scale with established diagnostic capabilities, designed to evaluate cognitive performance regarding an individual’s functioning in everyday tasks, with an average global score ranging from 0, indicating no impairment, to 3, indicating severe disability. Exclusion criteria for the Hillblom Aging Network cohort includes a CDR above 0, and/or a diagnosis of dementia, MCI, other neurological conditions, or severe mental or psychiatric disorders (such as stroke, brain tumor, schizophrenia, major depressive disorder, active substance use disorder, or bipolar disorder).

**Table 1:** Population demographics.

	Mean (SD or range) or Count (%)
Sample size	212
Number of observations	320
Female	108 (51%)
Average number of visits	1.5 (1–3)
Age	73.3 (6.2)
Years education	17.4 (2.2)
PASE scores	126.2 (64.1)

### Physical Activity

Physical activity was measured using the Physical Activity Scale for the Elderly (PASE), a validated measure of self-reported activity levels for the geriatric population. This measure was developed in older adults and asks participants to describe their physical activity engagement over 7 days, including questions aimed to assess duration, frequency, exertion level, and amount of physical activity. A composite score was calculated according to the scoring manual (Washburn et al., 1993). PASE scores in the current sample ranged from 0 to 361 (possible scores range 400+), with higher scores indicating higher levels of physical activity.

### Neuroimaging

#### MRI acquisition

Subjects were scanned at the UCSF Neuroscience Imaging Center on a Siemens Trio 3T scanner. A T1-weighted MP-RAGE structural scan was acquired with an acquisition time = 8 min 53 s, sagittal orientation, a field of view of 160 × 240 × 256 mm with an isotropic voxel resolution of 1 mm^3^, TR = 2,300 ms, TE = 2.98 ms, TI = 900 ms, flip angle = 9°. Task-free T2*-weighted echoplanar fMRI scans were acquired with an acquisition time = 8 min 06 s, axial orientation with interleaved ordering, the field of view = 230 × 230 × 129 mm, matrix size = 92 × 92, effective voxel resolution = 2.5 × 2.5 × 3.0 mm, TR = 2,000 ms, TE = 27 ms, for a total of 240 volumes. During the 8-min tf-fMRI acquisition protocol, participants were asked to close their eyes and concentrate on their breath.

#### fMRI Preprocessing

fMRI processing and network construction has been previously described in greater detail (Ashburner, 2007; Ashburner and Ridgway, 2013; Staffaroni et al., 2018). For each fMRI scan, the first five volumes were discarded. SPM12^1^ and FSL^2^ software were used for subsequent fMRI preprocessing (Ashburner and Friston, 2005; Jenkinson et al., 2012). The remaining 235 volumes were slice-time corrected, realigned to the mean functional image and assessed for rotational and translational head motion. Volumes were next co-registered to the MP-RAGE image, then normalized to the standard MNI-152 healthy adult brain template using the SPM segment, producing MNI-registered volumes with 2mm^3^ isotropic resolution. These volumes were spatially smoothed with a 6-mm radius Gaussian Kernal and temporally bandpass filtered in the 0.008–0.15 Hz frequency range using fslMaths. Nuisance parameters in the preprocessed data were estimated for the cerebrospinal fluid (CSF) using a mask in the central portion of the lateral ventricles and for the white matter using a mask of the highest probability cortical white matter as labeled in the FSL tissue prior mask. Additional nuisance parameters included the three translational and three rotational motion parameters, the temporal derivatives of the previous eight terms (WM/CSF/6 motion), and the squares of the previous 16 terms (Satterthwaite et al., 2013). Subjects were included only if they met all of the following criteria: no inter-frame head translations greater than 3 mm, no inter-frame head rotations greater than 3°, and less than 24 motion spikes (defined as inter-frame head displacements > 1 mm), 10% of the total frames.

Regions with insufficient fMRI BOLD signal to noise ratio were excluded using a previously described procedure (Staffaroni et al., 2018). Based on this procedure, we dropped nine scans and 45 nodes (which were excluded before deriving network metrics). Those excluded nodes that were part of the DMN networks were: left frontal medial cortex (47), right frontal pole (48), left inferior (95) and middle (81) temporal gyri, posterior divisions. Excluded nodes from the frontoparietal network included posterior, temporal gyrus (99, 101), the right frontal pole (46), and right inferior temporal gyrus (100, 102), and cerebellar regions (255, 258). Numbers in parentheses correspond to the nodes in the Brainnetome atlas (Fan et al., 2016). No subcortical nodes were excluded.

#### Network Construction

Functional networks were defined in a data-driven fashion using a set of 75 healthy older adult control subjects (our “Hillblom Aging Network” group; mean age = 65.3 ± 10.0 years, 33 females/42 males, mean education = 17.3 ± 2.1 years, 68 right handed/7 left-handed), scanned and analyzed using the same pipeline as the subjects in the longitudinal portion of this study. The details of our process have been described elsewhere (Staffaroni et al., 2018). Briefly, we utilized a modularity-based method for identifying which nodes comprised each module or “intrinsic connectivity network,” adopting a strategy that is conceptually similar to that used by Power et al. (2011), implementing the Brain Connectivity Toolbox^3^. Of the 418 time points for Hillblom Aging Network controls with PASE data and MRI scans, 341 scans were included in the primary analysis. 18 were removed pre-resting state (due to missing imaging acquisitions, sleep, scanner issues, or abnormalities such as temporal lobe cysts), 16 were removed pre-processing (due to coregistration failure, melodic failure, T1/coregistration failure, or missing data), 34 were removed due to motion, and nine were removed due to insufficient BOLD signal as described above. In Hillblom subjects, we determined the whole-brain functional connectome using 228 regions from the Brainnetome atlas (Fan et al., 2016). Networks analyzed in this study included the DMN, Frontoparietal, and Subcortical networks. For each participant, we calculated four mean FC values by taking the mean of the edges between all nodes within (Intra-) or between networks: (1) Intra-DMN; (2) Intra-Frontoparietal; (3) Intra-Subcortical; and (4) Cortico-Subcortical (between subcortical and frontoparietal networks).

#### Diffusion Tensor Imaging (DTI)

Our DTI pipeline has been described previously (Elahi et al., 2017). Briefly, FSL software (Jenkinson et al., 2012) was used to co-register the diffusion direction images with the *b* = 0 image, then a gradient direction eddy current and distortion correction were applied. Diffusion tensors were calculated using a non-linear least-squares algorithm from Dipy (Garyfallidis et al., 2014). After quality control, participants’ tensors were registered linearly and non-linearly into a common space using DTI-TK (Zhang et al., 2006).

### Neuropsychological Outcomes

#### Composite Cognitive Measures

Composite measures were used to summarize neuropsychological performance across domains. *Episodic memory* was assessed using the Benson Figure Recall (Kramer et al., 2003), a measure of visual memory, and subscores of the California Verbal Learning Test, second edition (CVLT-II; Delis et al., 2000): immediate recall total, long (20 min) delay free recall total, and recognition discriminability (d’). *Executive functions* were evaluated with the Stroop interference test (Stroop, 1935), modified Trail Making Test (Kramer et al., 2003), lexical fluency (D-words/min; Kramer et al., 2003), digit span backward (Wechsler, 1997), and Design Fluency (D-KEFS Condition 1; Delis et al., 2001). *Processing speed* was assessed using six computerized visuospatial processing speed tasks that have been previously described elsewhere (Kerchner et al., 2012; Casaletto et al., 2019).

### Cardiovascular Covariates

As part of the neurological examination, subjects completed vital measurements that have been identified as risk factors for dementia and may be indicative of brain health, including height and body weight [which were used to calculate body mass index (BMI)], resting heart rate, and resting blood pressure.

### Brain Structure Covariates

To determine the relative independence of the relationship between physical activity and fMRI connectivity from white matter changes (as a proxy for cerebrovascular health) or gray matter atrophy in the queried networks, we then extracted fractional anisotropy (FA), mean diffusivity (MD), and gray matter volume from the specific networks of interest. Based on known anatomical relationships, DTI-based white matter tracts were extracted for: *subcortical network*: anterior limb of the internal capsule and the posterior thalamic radiation; *executive-subcortical network*: anterior limb of the internal capsule, posterior thalamic radiations, superior longitudinal fasciculus, genu, body, and splenium of the corpus callosum. The Brainnetome regions comprising the tf-fMRI networks (subcortical and executive-subcortical) were then applied to the T1 scans to extract gray matter volumes. The regional volumes for all regions comprising a given network were summed to obtain the network’s gray matter volume.

### Statistical Analyses

First, we fitted baseline cross-sectional multivariable linear regression models examining the relationship between PASE scores and FC in our networks of interest, covarying for demographic factors (age, sex, years of education). Parallel models examined the relationship between PASE scores and neuropsychological test performances, again adjusting for age, sex, and years of education. Our primary analyses focused on our three networks of interest (the DMN, the FPN, and the SN). Then, in the networks that reached statistical significance, we conducted secondary analyses to examine their inter-network relationships, to better understand the associations at play.

Next, we conducted linear mixed-effects models to examine the longitudinal relationship between changes in PASE scores and FC trajectories in the networks of interest, as well as between PASE scores and cognitive performance. In all models, following (Neuhaus and Kalbfleisch, 1998; Neuhaus and McCulloch, 2006), we decomposed PASE scores into within- (i.e., change per visit) and between- (i.e., average) subject effects to associate purely within-subject changes in physical activity with changes in fMRI and cognitive outcomes, as well as to avoid estimation bias resulting from incorrectly assuming common within- and between-subject effects.

In our first longitudinal model, we examined how between- and within-subject changes in PASE scores related to changes in FC, adjusting for demographic factors (sex, age and years of education), as well as markers of cardiovascular health (time-varying heart rate, BMI and systolic reading) as covariates. Models in which networks significantly tracked with changes in PASE scores (at *p* < 0.05) were selected for follow-up analyses, to explore whether this relationship upheld after adjusting for markers of cerebrovascular integrity. For this purpose, we conducted parallel models covarying for gray matter volume and FA, or gray matter volume and MD in the gray matter ROIs and white matter tracts associated with the given network of interest. We re-scaled predictors to standard deviation units, in concordance with standardized beta coefficients, as seen in Table 2. Lastly, we examined the longitudinal relationship between within- and between-person increases in PASE scores with neuropsychological trajectories (separate models per cognitive composite).

**Table 2:** PASE scores and Functional Connectivity after controlling for markers of cardiovascular health (Model 1; *N* = 179) and cerebrovascular health (Model 2; *N* = 175).

	Intra subcortical				Inter executive-subcortical			
	Beta Coefficient	Lower 95% CI	Upper 95% CI	*P* > |*z*|	Beta Coefficient	Lower 95% CI	Upper 95% CI	*P* > |*z*|
**Model 1**								
Age	0.003	−0.020	0.025	0.797	−0.008	−0.031	0.080	0.374
Education	0.047	−0.018	0.111	0.158	0.060	−0.006	0.125	0.073
Sex	0.027	−0.259	0.313	0.852	−0.086	−0.375	0.203	0.559
Blood	0.002	−0.005	0.009	0.0560	0.002	−0.005	0.009	0.595
pressure	−0.014	−0.025	−0.002	0.017	−0.007	−0.019	0.004	0.220
Heart rate	0.021	−0.011	0.053	0.205	−0.005	−0.037	0.027	0.764
BMI								
Within-subject PASE	**0.329**	**0.090**	**0.569**	**0.007**	**0.266**	**0.023**	**0.509**	**0.032**
Between-subject PASE	−0.042	−0.187	0.103	0.573	−0.066	−0.212	0.080	0.374
**Model 2**								
Age	−0.002	−0.026	0.022	0.872	−0.010	−0.035	0.015	0.434
Education	0.042	−0.023	0.107	0.201	0.059	−0.007	0.124	0.081
Sex	0.014	−0.299	0.327	0.929	−0.112	−0.431	0.207	0.493
Blood	0.0001	−0.007	0.007	0.971	0.0002	−0.007	0.008	0.960
pressure	−0.013	−0.025	−0.001	0.032	−0.007	−0.019	0.006	0.005
Heart rate	0.023	−0.009	0.056	0.162	−0.0001	−0.033	0.033	0.995
BMI
Network FA	−1.358	−5.378	2.661	0.508	−2.647	−6.581	1.286	0.187
Subcortical	0.00001	−0.00009	0.0001	0.824	−0.00009	−0.0002	0.00005	0.187
GMV	—	—	—	—	0.00002	−0.000001	0.00004	0.151
Exec GMV								
Within-subject PASE	**0.313**	**0.071**	**0.554**	**0.011**	**0.277**	**0.023**	**0.531**	**0.032**
Between-subject PASE	−0.043	−0.190	0.103	0.562	−0.060	−0.208	0.088	0.428

*Note. Network FA/MD/GMV reflects either striatal or frontal-striatal structures for the intra subcortical and inter executive-subcortical FC models, respectively. Abbreviations: BMI, body mass index; PASE, Physical Activity Scale for the Elderly; FA, fractional anisotropy; MD, mean diffusivity. The significance of bold values is p< 0.05*.

## Results

### Physical Activity Level, FC, and Cognition at Baseline Demonstrate Small, Non-statistically Significant Associations

At baseline, reported physical activity levels demonstrated weak negative relationships with FC in brain regions of interest: intra-DMN network connectivity (*β* = −0.09, *p* = 0.20), intra-executive network connectivity (*β* = −0.12, *p* = 0.08), intra-subcortical network connectivity (*β* = −0.05, *p* = 0.51), and executive-subcortical inter-network connectivity (*β* = −0.07, *p* = 0.35). Similarly, the cross-sectional relationship between physical activity and cognitive performance was small and nonsignificant (processing speed *β* = 0.01, *p* = 0.92; executive functioning *β* = −0.06, *p* = 0.38; and memory *β* = −0.01, *p* = 0.91).

### Longitudinal Increases in Reported Physical Activity Relate to Increases in FC, Independent of Demographics and Cardiovascular Health Markers

**Figure 1:**
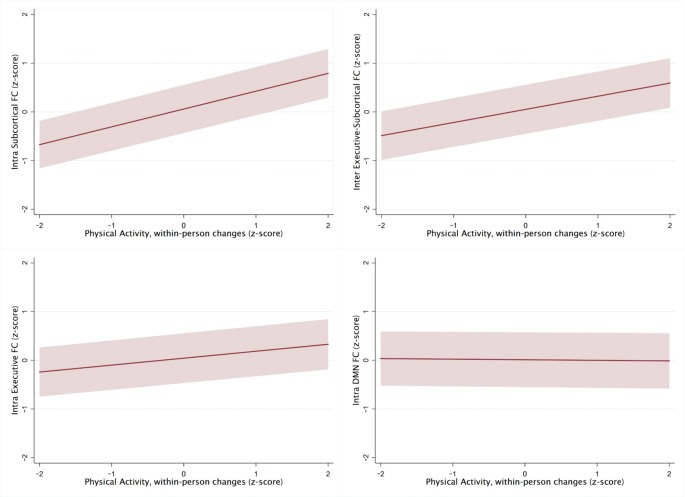
Within-person changes in Physical Activity Scale for the Elderly (PASE) scores in ROIs, controlling for demographic and cardiovascular health factors.

Adjusting for age, sex, education, and cardiovascular health, within-subject increases in PASE scores, but not overall between-person PASE, tracked with greater intra-subcortical network (PASE within-subject: *β* = 0.33, *p* = 0.007; PASE between-subject: *β* = −0.04, *p* = 0.57) and frontal-subcortical inter-network (PASE within-subject: *β* = 0.27, *p* = 0.03; PASE between-subject: *β* = −0.07, *p* = 0.37) synchrony over time on fMRI. Neither within-person nor between-person changes in PASE scores were significantly related to DMN (PASE within-subject: *β* = −0.02 *p* = 0.89; PASE between-subject: *β* = −0.05, *p* = 0.49) or intra-frontoparietal network (PASE within-subject: *β* = 0.15, *p* = 0.23; PASE between-subject: *β* = −0.11, *p* = 0.15) connectivity (see Table 2, Figure 1).

### Longitudinal Increases in Reported Physical Activity and Greater tf-fMRI Connectivity Relationship Upheld After Controlling for Network-Specific White Matter Microstructure and Gray Matter Volume

Additionally, adjusting for FA and gray matter in the networks that demonstrated significant longitudinal relationships with PASE demonstrated the same pattern of results, with similar effect sizes. Within-subject increases in PASE scores tracked with greater intra-subcortical network synchrony (*β* = 0.31, *p* = 0.01), while between-subject effects continued to be small and did not reach statistical significance (*β* = −0.04, *p* = 0.56). Similarly, within-subject increases in PASE scores tracked with greater inter-executive-subcortical network synchrony (*β* = 0.28 *p* = 0.03), yet between-subject effects were small and did not reach statistical significance (*β* = −0.06, *p* = 0.43). Moreover, we examined parallel models adjusting for MD and gray matter and observed the same pattern as when controlling for FA (see Table 2).

### PASE Scores and Cognitive Outcomes Did Not Show Statistically Significant Longitudinal Associations in Typically Aging Adults

Both within- and between-subject self-reported levels of physical activity demonstrated small, nonsignificant associations with cognitive performance (within-subjects β range = −0.14 to 0.002, *p*s < 0.98; between-subjects β range = −0.04 to 0.008, *p*s < 0.93).

## Discussion

### Summary

This study examined the longitudinal relationship between physical activity and FC in different regions of the brain. We found that specific within-person increases in physical activity may track closely with FC. Importantly, there appears to be specificity regarding the regionality of this effect. Our findings suggest that within-person increases in physical activity are specifically associated with greater frontal-subcortical and within-subcortical network synchrony. Increased FC in these networks may further support the positive effect of physical activity on brain health markers and adds to this literature by suggesting that within-person augmentation of personal exercise regimes may relate to within-person brain changes, regardless of activity level or cognitive status at baseline.

Additionally, and consistent with previous literature, in our study, FC in the DMN did not appear to be affected by increases in physical activity. While the DMN has received the most attention in fMRI studies, given its important role in the development of age-related neurodegenerative processes (i.e., Alzheimer’s disease; Biswal et al., 2010), this network appears to be less importantly related to brain-exercise benefits, but it has historically limited the focus on alternative networks, including subcortical processes. The subcortical network is formed by a group of structures that are vastly connected with the rest of the brain. This network has been described to be responsible for highly advanced motor tasks and movement coordination (Fama and Sullivan, 2015). Likewise, the FPN is accountable for goal-driven, organized and controlled execution of behaviors (Marek and Dosenbach, 2018). Both networks are heavily involved in executive processes, suggesting that FC, improved by physical activity engagement, could ultimately act as a protective factor for executive function in typically aging older adults. Although our results do not demonstrate a strong relationship between self-reported physical activity and longitudinal cognitive trajectories, particularly in those domains related to executive functioning and processing speed, there is still exhaustive work to be done to explore this possibility more in-depth. Possibly our current neuropsychological battery is not sensitive enough to detect changes in cognition that are related to the observed augmentation of network synchronicity. Alternatively, the characteristics of our sample (well-educated, high socioeconomic status) could have affected the expression of these changes during cognitive testing. The weak association between physical activity and cognition in our results may be due to our cohort’s high level of cognitive function, as previous literature suggests that exercise effects are more potent in cognitively impaired individuals (Sanders et al., 2019). Therefore, it would be prudent to study how these results would vary if examined in populations that are more vulnerable to age-related brain changes, where changes in cognitive trajectories and expressions of these changes may be more variable. That being said, the fact that our data demonstrate a significant association between FC and increased physical activity, even in a cognitively-intact and highly-functional cohort, may be particularly robust.

Our findings are aligned with previous literature in the field that has shown a link between physical activity and brain health (Daskalopoulou et al., 2017; Macpherson et al., 2017). On the one hand, scientists are becoming increasingly interested in the search for a harmonized definition and breakdown of what physical activity represents, the optimal quantity required for salubrious effects, and a standardized unit of measurement. On the other hand, as we move towards a precision medicine approach, we may realize there is no universal recipe for physical activity, and instead fully embrace the multiple benefits that exercise offers us.

Similarly, the benefits of physical activity are likely not attributable to a single mechanism, but a wide range of biological changes within the body at a multiorgan level. Scientists have examined this hypothesis mostly in animal models, describing for example how exercise increases brain-derived neurotrophic factor (BDNF) levels (Pedersen, 2019). Synaptic growth seems to be another plausible effect of physical activity on FC, which could be playing a role in our results as well. Our findings are therefore especially novel in demonstrating the importance of frontal and subcortical networks in exercise. Furthermore, by showing a longitudinal relationship between physical activity and functional connectivity that is independent of cardiovascular and cerebrovascular factors, we are opening the door to better understand how physical activity may change brain integrity on a cellular level.

### Limitations

Although this study sheds light on the possible neural networks by which physical activity affects the brain, there are some notable limitations. Firstly, there may be limitations regarding the generalizability of our results, as this study consists of a convenience sample that may be affected by selection bias regarding the type of individual that decides to participate in research. Of course, this is a limitation that spans across the majority of research projects (Hedt and Pagano, 2011). The UCSF Memory and Aging Center’s Community Outreach Program is aiming to address this issue by engaging with the San Francisco community and encouraging participation among more diverse populations. Given that data were pulled from an ongoing, longitudinally-followed cohort, as we continue data collection, we plan to continue to examine these questions in larger, more demographically- and clinically-diverse samples with greater timepoint accrual. Still, ours is one of the first studies to examine the longitudinal relationship between exercise and neural networks in older adults, and it represents an important first step in understanding these relationships.

Another possible limitation lies in the use of self-reported measures of exercise, which may be biased due to factors such as measurement error (Butler et al., 1987), social desirability/recall bias (Adams et al., 2005), or longitudinal response-shift bias (Rosenman et al., 2011). That being said, the PASE is a widely-used measure of physical activity that has been previously validated through comparison to both indirect and direct measures of physical activity (Logan et al., 2013).

Finally, although the current study used longitudinal data to examine within-person variability in physical activity and FC, due to the utilization of an observational study design, we cannot determine causality. Still, our within-subject findings provide compelling support for the tight coupling between the brain and exercise behaviors. To fully disentangle directionality, future research should examine within- and between-person changes in brain synchrony through the utilization of an interventional study design across extended longitudinal timepoints.

### Future Directions

Further investigation of various physical activity behaviors is critical to improving the brain health outcomes of the growing aging population. We and others are expanding work on objective actigraph-based activity data to ameliorate the potential self-report bias effects (Spartano et al., 2019). Continued work implementing experimental physical activity interventions would allow us to determine causality in the relationship between physical activity and FC, and thus give us further support for directionality and the mediating role of brain synchrony in the relationship between physical activity and brain health.

Regarding clinical practices, our findings suggest that it may be worthwhile for clinicians to encourage even incremental increases in physical activity, to benefit brain network functioning. This may be of particular importance to individuals manifesting symptoms involving the frontal-subcortical networks that we identified as related to physical activity, such as cerebrovascular or Parkinson’s disease (Zhu et al., 2019). Going forward, there is a clear need for more prospective human studies examining the benefits of various types and quantities of exercise, as well as the motivational aspects of encouraging increased physical activity in older adults. We plan to work on disentangling the contributions of exercise intensity, frequency, and duration, while also looking into the relationship between brain connectivity and markers of inflammation.

## Data Availability Statement

All data obtained and processed through this project are part of the UCSF Memory and Aging Center data infrastructure. All data collected are available for sharing with appropriate approval from the UCSF Human Subject Protection committee, Health Insurance Portability and Accountability Act regulations, and all applicable state and federal laws. All data requests are processed through the UCSF Alzheimer’s Disease Research Center Administrative Core, where applicants complete an online application where they provide a research rationale and evidence of regulatory approvals.

## Ethics Statement

The studies involving human participants were reviewed and approved by UCSF Human Research Protection Program & IRB. The patients/participants provided their written informed consent to participate in this study.

## Author Contributions

Project conception, design, and statistical analyses were completed by KC, KD, and AS. As co-first authors, KD and SW-L completed the literature review and wrote the manuscript. Select methodological sections were conceived and drafted by YC, JB, AW, and AS (network construction, fMRI processing). SW and AW worked on neuroimaging processing and database cleaning. JK and KC supervised the project and provided critical edits of the manuscript.

## Conflict of Interest

The authors declare that the research was conducted in the absence of any commercial or financial relationships that could be construed as a potential conflict of interest.

## Footnotes

^1^http://www.fil.ion.ucl.ac.uk/spm/software/spm12/

^2^http://fsl.fmrib.ox.ac.uk/fsl

^3^https://sites.google.com/site/bctnet/
